# In maize, co-expression of GAT and GR79-EPSPS provides high glyphosate resistance, along with low glyphosate residues

**DOI:** 10.1007/s42994-023-00114-8

**Published:** 2023-09-01

**Authors:** Shengyan Li, Pengcheng Li, Xiangyin Li, Ning Wen, Yinxiao Wang, Wei Lu, Min Lin, Zhihong Lang

**Affiliations:** 1 Biotechnology Research Institute, Chinese Academy of Agricultural Sciences, Beijing, China; 2 National Nanfan Research Institute (Sanya), Chinese Academy of Agricultural Sciences, Sanya, Hainan China

**Keywords:** Transgenic maize, Glyphosate resistance, *Gr79-epsps* gene, *Gat* gene, Low residues

## Abstract

Herbicide tolerance has been the dominant trait introduced during the global commercialization of genetically modified (GM) crops. Herbicide-tolerant crops, especially glyphosate-resistant crops, offer great advantages for weed management; however, despite these benefits, glyphosate-resistant maize (*Zea mays* L.) has not yet been commercially deployed in China. To develop a new bio-breeding resource for glyphosate-resistant maize, we introduced a codon-optimized glyphosate N-acetyltransferase gene, *gat*, and the enolpyruvyl-shikimate-3-phosphate synthase gene, *gr79-epsps*, into the maize variety B104. We selected a genetically stable high glyphosate resistance (GR) transgenic event, designated GG2, from the transgenic maize population through screening with high doses of glyphosate. A molecular analysis demonstrated that single copy of *gat* and *gr79-epsps* were integrated into the maize genome, and these two genes were stably transcribed and translated. Field trials showed that the transgenic event GG2 could tolerate 9000 g acid equivalent (a.e.) glyphosate per ha with no effect on phenotype or yield. A gas chromatography-mass spectrometry (GC–MS) analysis revealed that, shortly after glyphosate application, the glyphosate (PMG) and aminomethylphosphonic acid (AMPA) residues in GG2 leaves decreased by more than 90% compared to their levels in HGK60 transgenic plants, which only harbored the *epsps* gene. Additionally, PMG and its metabolic residues (AMPA and N-acetyl-PMG) were not detected in the silage or seeds of GG2, even when far more than the recommended agricultural dose of glyphosate was applied. The co-expression of *gat* and *gr79-epsps*, therefore, confers GG2 with high GR and a low risk of herbicide residue accumulation, making this germplasm a valuable GR event in herbicide-tolerant maize breeding.

**Supplementary Information:**

The online version contains supplementary material available at 10.1007/s42994-023-00114-8.

## Introduction

Maize (*Zea mays* L.) has been domesticated for more than 9000 years and is a leading cereal crop for food, feed, industrial products, and energy production. The global demand for maize is predicted to nearly double between 2020 and 2050, due to economic development and population growth (Erenstein et al. 2022). China is the second largest producer of maize in the world, with increases in its planted acreage and yield of nearly 170 percent and 228 percent, respectively, over the past 20 years (NBSC 2021). Despite these increases, the demand for maize is growing each year, and the shortfall for this crop is predicted to reach as much as 66.24 billion kilograms (kg) by 2050, with a self-sufficiency rate estimated at 82% (Huang et al. 2022).

Weeds are wild plants that compete with crops for water, light, soil nutrients, and space, and can also become hosts for various plant pests and diseases. In China, about 92.47 million hectares (ha) of farmland are affected by weeds, including 26.67 million ha of maize fields, which corresponds to about 50% of the maize planting area, resulting in an annual crop yield loss of more than 30 billion kg (Li 2018). Weed control is therefore essential to achieving optimal crop yields.

Chemical control (using herbicides) is the most economical and effective weed management strategy. Glyphosate [N-(phosphonomethyl)-glycine] is a highly effective broad-spectrum herbicide that is highly phytotoxic but has low toxicity to fauna (Duke 2018; Duke and Powles 2008; Woodburn 2000). Glyphosate can compete with phosphoenolpyruvate (PEP) for binding to 5-enolpyruvylshikimate-3-phosphate synthase (EPSPS), blocking the shikimate pathway and resulting in plant death (Dill 2005; Fuchs et al. 2021). Due to its non-selective characteristics, glyphosate kills weeds and crops alike. To confer glyphosate resistance (GR) to transgenic crops, two strategies are usually employed. The first method is to heterologously express exogenous or mutant version of *epsps*, encoding enzymes that are insensitive to glyphosate. Examples of such GR *epsps* genes include *cp4-epsps* from *Agrobacterium tumefaciens* CP4 (Meilan et al. 2002; Ridley et al. 2002), *g2-epsps* from *Pseudomonas fluorescens* G2 (Dun et al. 2007), *g6-epsps* from *Pseudomonas putida* (Zhao et al. 2011), *g10evo* from *Deinococcus radiodurans* R1 (Peng et al. 2021), and I. variabilis-epsps* from *Isoptericola variabilis* (Cui et al. 2016). In addition, some GR genes have been engineered from plant *epsps* genes via amino acid substitution, such as *Zm-mepsps* from maize (Sidhu et al. 2000); *Os-mepsps*, *TIPS-Osepsps,* and *GATIPS-Osepsps* from rice (*Oryza sativa* L.) (Achary et al. 2020; Chandrasekhar et al. 2014); *Mdepsps* from apple (*Malus domestica*) (Tian et al. 2013); and *TIPS-Eiepsps* from Indian goosegrass (*Eleusine indica*) (Yu et al. 2015). Among these, only *cp4-epsps* and *Zm-mepsps* genes are currently deployed in commercial GM products (Ridley et al. 2002; Sidhu et al. 2000).

The second strategy to confer GR to transgenic crops is to express genes that encode functional enzymes that can modify or degrade glyphosate, such as glyphosate *N*-acetyltransferase (*gat*) and glyphosate oxidoreductase (*gox*). *Gat* was originally isolated from *Bacillus licheniformis*, encoding an enzyme that acetylates glyphosate to *N*-acetyl-glyphosate. The *gat4601* and *gat4621* genes have been functionally improved, via gene-shuffling, to optimize GAT kinetics, and have been applied in commercial GM soybean (*Glycine max*), rapeseed (*Brassica napus*), and maize (Castle et al. 2004; Siehl et al. 2005, 2007). The *gox* gene, isolated from *Ochrobactrum anthropi* strain LBAA, encodes an enzyme that can degrade glyphosate to glyoxylate and aminomethylphosphonic acid (AMPA) (Dill 2005; Tan et al. 2006). Transgenic plants harboring the *gox* gene alone are not able to achieve commercial-level GR, partly due to AMPA phytotoxicity (Feng et al. 2010; Reddy et al. 2004). Only *goxv247*, a modified *gox* gene with three amino acid substitutions, has been effectively applied in commercial rapeseed products. Although the overexpression of *epsps* can result in a high level of GR in plants, tissue-specific gaps in its expression can also lead to glyphosate injury (Feng et al. 2010). GAT can acetylate glyphosate to remove its residue from the plant. The resulting product *N*-acetyl-glyphosate is not herbicidal as it is not an effective inhibitor of EPSPS (Castle et al. 2004), indicating that the co-expression of *epsps* and *gat* may offer an improved strategy for developing glyphosate-resistant crops.

The *gat* and *gr79-epsps* genes were initially isolated from an extremely glyphosate-polluted soil (Dun et al. 2006; Jin et al. 2007). Previous studies showed that transgenic tobacco (*Nicotiana tabacum* L.) expressing *gat* or *gr79-epsps* alone was highly resistant to glyphosate (Cao et al. 2012; Dun et al. 2014). Furthermore, upland cotton (*Gossypium hirsutum*) co-expressing *gat* and *gr79-epsps* had a higher GR than cotton expressing only one of these GR genes (Liang et al. 2017). In this study, we developed a highly glyphosate-resistant transgenic event in maize, designated GG2, by co-expressing *gat* and *gr79-epsps*. We show that GG2 contains low levels of glyphosate residues and has significant resistance to higher doses of glyphosate under field conditions. Furthermore, the agronomic traits of GG2 plants were comparable to those of the non-transgenic control. The transgenic maize event GG2 has passed the pre-production testing, is food safe, and poses no environmental risk. This study is a major step forward for the commercial production of new herbicide-resistant maize.

## Results

### Maize transformation and screening for high GR

**Fig. 1:**
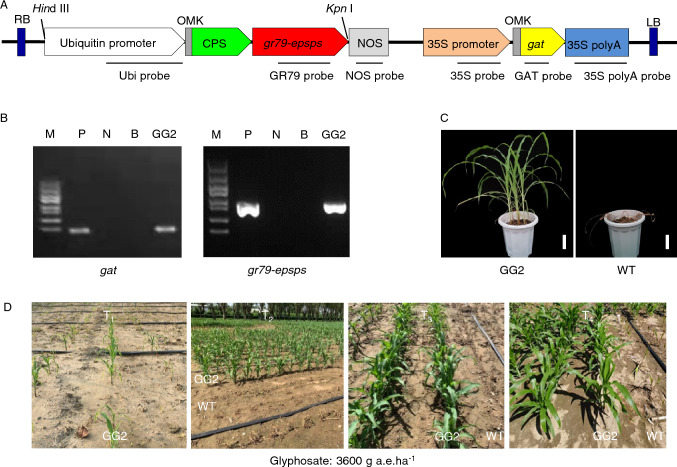
Construction of the plant expression cassette, molecular detection, and glyphosate screening of the transgenic maize plants. **A** Diagram of the expression cassette. The *gr79-epsps* and *gat* genes were expressed under the maize *Ubiquitin* promoter and CaMV 35S promoter, respectively. *OMK* synthetic Omega and Kozak sequences to enhance gene expression, *CPS* chloroplast peptide signal sequence from the maize ribulose bisphosphate carboxylase small subunit. The indicated probes were used in a Southern blot analysis. **B** PCR screening of GG2 plants with the *gat* and *gr79-epsps* primers. *M* Trans5K DNA Marker, *P* positive control pCGG vector, *N* wild-type maize plants, *B* blank. **C** T_1_ transgenic maize event GG2 showing high resistance to a 3600 g a.e. ha^–1^ glyphosate application in the greenhouse. Scale bars, 10 cm. **D** Transgenic maize event GG2 showing high resistance to a 3600 g a.e. ha^–1^ glyphosate application in successive generations of field trials

To express *gat* and *gr79-epsps* to high levels in maize, we codon-optimized the sequences of both genes using the OptimumGene algorithm and synthesized the resulting sequences (Figure S1 and S2). The codon adaptation index (CAI) values of the original *gat* and *gr79-epsps* genes for maize were 0.70 and 0.60, respectively, with GC contents of 47.9% and 45.85%. After optimization, the CAIs of the two genes increased to 0.94 and 0.93, respectively, and their GC contents changed to 63.9% and 64.6%. We constructed the vector pCGG containing the *gat* and *gr79-epsps* expression cassettes and transferred the resulting clone into immature embryos of maize variety B104 using *Agrobacterium* (*Agrobacterium tumefaciens*)-mediated transformation (Fig. 1A). The efficiency of transformation was approximate 10%. We subjected the regenerated plants to PCR analysis for the transgene, retaining only those containing both *gat* and *gr79-epsps* for GR screening. We planted the T_1_ generation of positive transformants in the field and sprayed them with a high dose of glyphosate (3600 g acid equivalent [a.e.] glyphosate ha^–1^) at the four-leaf stage. We self-pollinated the surviving glyphosate-resistant plants and outcrossed them to the elite inbred lines Zheng58 and Chang7-2. We ultimately selected the independent transgenic maize event GG2 from the regenerated maize population based on PCR screening and high-dose glyphosate testing over several generations (Fig. 1B). The plants of transgenic event GG2 harboring the *gat* and *gr79-epsp*s genes grew normally under a 3600 g a.e. ha^–1^ glyphosate treatment in the greenhouse and field (Fig. 1C, D). The approximate resistant/sensitive ratio in the F_1_ generation of GG2 backcrosses was 1:1, which was consistent with Mendelian segregation for an insertion of the T-DNA at a single site (Table S1). We obtained the homozygous transgenic event GG2 in the B104 genetic background through the continuous selfing of the T_0_ generation, while we introgressed the homozygous transgenic event GG2 into the Zheng58 (Z58) and Chang7-2 (C7-2) backgrounds through continuous backcrossing for six generations from the T_1_ generation and then selfing for three generations. We also introgressed GG2 into a Zhengdan958 (ZD958) hybrid background by crossing GG2 (Z58) to GG2 (C7-2). The breeding pedigree of the transgenic event GG2 is shown in Figure S3.

### Molecular analysis of the transgenic maize event GG2

**Fig. 2:**
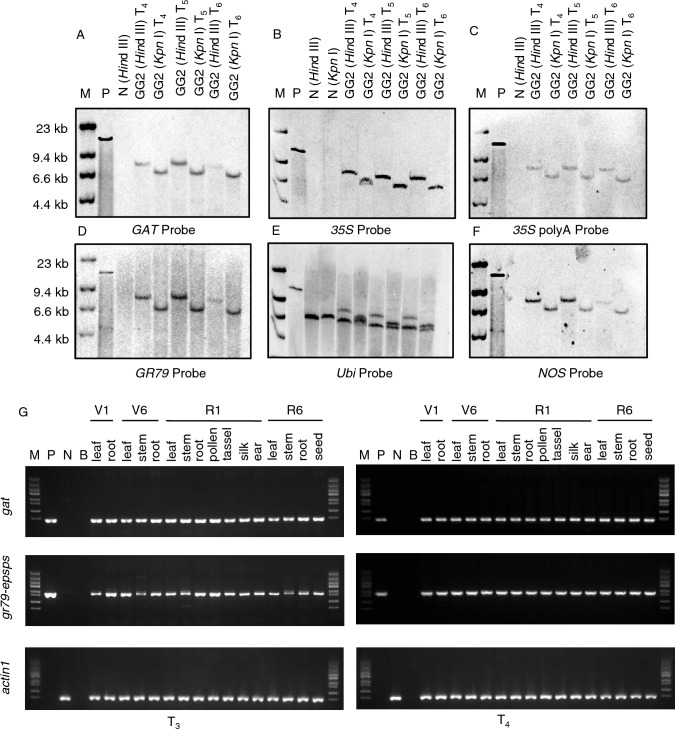
Southern blot and end-point RT-PCR analyses of the *gat* and *gr79-epsps* copy number and expression in transgenic maize event GG2. **A**–**F** Southern blots using probes for the *gat* gene, the 35S promoter, the 35S polyA terminator, the *gr79-epsps* gene, the *Ubiquitin* promoter, and the *nos* terminator to determine the copy number in the T_4_–T_6_ generations of GG2 plants. *M* DIG-labeled lambda/HindIII marker for the Southern blot; *P* pCGG vector digested by HindIII; *N* genomic DNA of wild-type non-transgenic maize plants. **G** Expression levels of *gat* and *gr79-epsps* in maize tissues at four development stages (V1, V6, R1, and R6) of the T_3_ and T_4_ generations of GG2 plants, determined using end-point RT-PCR. The maize *Actin1* gene was used as the control. *M* Trans5K DNA Marker, *P* positive control pCGG vector, *N* wild-type maize plants, *B* blank

**Fig. 3:**
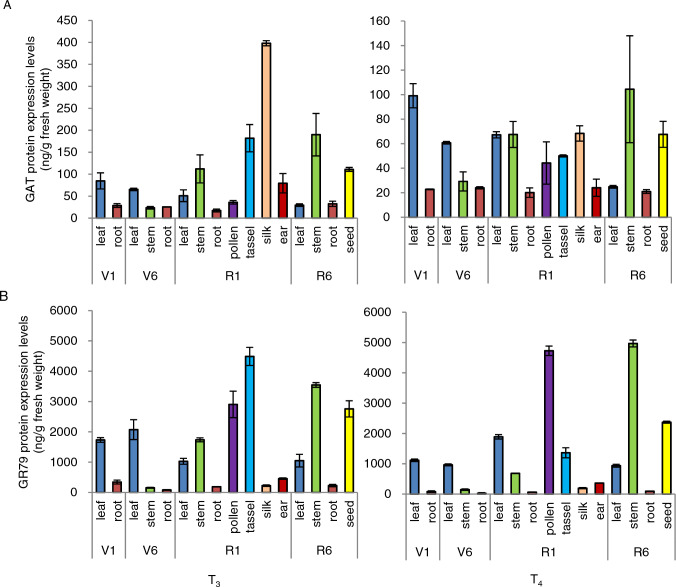
ELISA of GAT and GR79-EPSPS protein levels in the transgenic maize event GG2. Abundance of GAT (**A**) and GR79-EPSPS (**B**) in 16 maize tissues at four stages (V1, V6, R1, and R6) from the T_3_ and T_4_ generations of transgenic maize GG2 plants. Data are means ± SD (*n* = 3 biological replications)

We determined the genetic stability and copy number of the foreign expression cassettes in the transgenic maize event GG2 by Southern blot analysis with specific probes for the *gat* gene, 35S promoter, 35S polyA terminator, the *gr79-epsps* gene, the maize *Ubiquitin* promoter, and the *nos* terminator (Fig. 1A). We detected one hybridization band for all probes except the maize *Ubiquitin* promoter probe, which labeled the endogenous and transgenic copies of the promoter. We observed no signal from the hybridization of genomic DNA from wild-type (WT) plants with any probe, with the exception of the *Ubiquitin* probe (Fig. 2A–D and F). These Southern blot results suggest that the *gat* expression cassette and *gr79-epsps* expression cassette are stably integrated into the maize genome as single-copy insertions. Furthermore, we used three probes designed to cover the entire vector backbone to check whether it might be inserted into the maize genome (Figure S4a); importantly, we detected no signal, indicating that the vector backbone is not present in the genome of the GG2 event (Figure S4b–d). We confirmed the successful expression of *gat* and *gr79-epsps* by end-point RT-PCR and the accumulation of the encoded proteins by enzyme-linked immunosorbent assay (ELISA) in the GG2 plants. We conclude that these genes can be correctly transcribed and translated in maize tissues at different developmental stages and that they are stably inherited in the T_3_ and T_4_ generations of GG2 plants (Figs. 2G and 3). All these results confirm that the *gat* and *gr79-epsps* genes are present as single-copy insertions and are stably transcribed and translated in the transgenic event GG2.

### Identification of the T-DNA insertion site and flanking sequence of GG2 in the maize genome

**Fig. 4:**
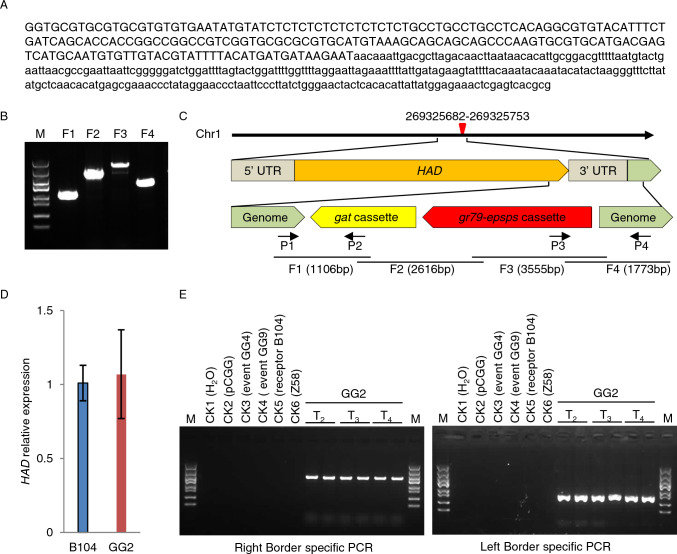
Identification of the T-DNA insertion site and flanking sequence of GG2 in the maize genome. **A** Sanger sequencing of the amplified TAIL-PCR product for the left border (LB) of GG2. The sequence of maize genomic DNA at the LB is denoted by capital letters and that of the T-DNA sequence by lowercase letters. **B** Amplification products of the expression cassette in GG2 plants using PCR. The locations and sizes of fragments F1, F2, F3, and F4 are shown in **C**. **C** Chromosomal location of the T-DNA sequence in GG2. The red triangle denotes the insertion site of the T-DNA. P1/P2 and P3/P4 are event-specific PCR primers for the LB- and RB-flanking sequence, respectively. **D** RT-qPCR analysis of relative *HAD* expression in the roots of B104 and GG2 (T_2_) plants. Data are means ± SD (*n* = 3 biological replications). *P* > 0.05, determined using Student’s *t*-test. **E** Event-specific PCR detection in the T_2_–T_4_ generations of GG2 plants

We determined the sequence flanking the T-DNA left border (LB) in the transgenic maize event GG2 using thermal asymmetric interlaced (TAIL)-PCR. We excised and purified the tertiary PCR product that was isolated from agarose gel and subjected it to Sanger sequencing, followed by a Basic Local Alignment Search Tool (BLAST) against the MaizeGDB (https://www.maizegdb.org, B73 RefGen_V4). Sequencing produced a 462-bp fragment for the GG2 insertion site, of which 194 bp originated from the maize genome and 268 bp originated from the T-DNA sequence (Fig. 4A). The LB insertion site is located on chromosome 1 at position 269,325,682 bp (B73_refgen_v4) of the maize genome. To make sure that the full-length expression cassettes were inserted, we designed four pairs of PCR primers (frag1-F/R, frag2-F/R, frag3-F/R, and frag4-F/R; Table S2) with partial overlap between individual PCR products to amplify four fragments (F1, F2, F3, and F4) covering the entire T-DNA sequence (Fig. 4B and C). We performed a BLAST search against the reference maize genome and assembled the sequences together when they overlapped: we discovered that the entire T-DNA sequence is inserted in reverse orientation relative to the direction of maize chromosome 1, between 269,325,682 bp and 269,325,753 bp (B73_refgen_v4). The T-DNA was located within the tail of the 3′ untranslated region (UTR) of the haloacid dehalogenase-like hydrolase gene (*HAD*), resulting in the deletion of a 47-bp sequence in the 3′ UTR and a 23-bp intergenic region sequence (Fig. 4C). A reverse-transcription quantitative PCR (RT-qPCR) analysis showed that *HAD* expression is not significantly different between the GG2 and B104 recipient plants (Fig. 4D), indicating that the insertion of the T-DNA in the GG2 plants does not affect *HAD* expression. To specifically detect and authenticate the transgenic event GG2, we designed two pairs of specific primers (P1/P2, P3/P4; Fig. 4C and Table S2) to amplify the extension of the LB- and RB-flanking sequences, in each case including part of the maize genome and part of the T-DNA. We detected the expected specific PCR products in GG2 plants but not in B104, Z58, or the other sister transgenic events (Fig. 4E). This result suggests that the two pairs of specific primers can be used to specifically detect and authenticate the transgenic event GG2 and its derivatives.

### Field evaluation of glyphosate resistance and agronomic traits of transgenic maize GG2

**Fig. 5:**
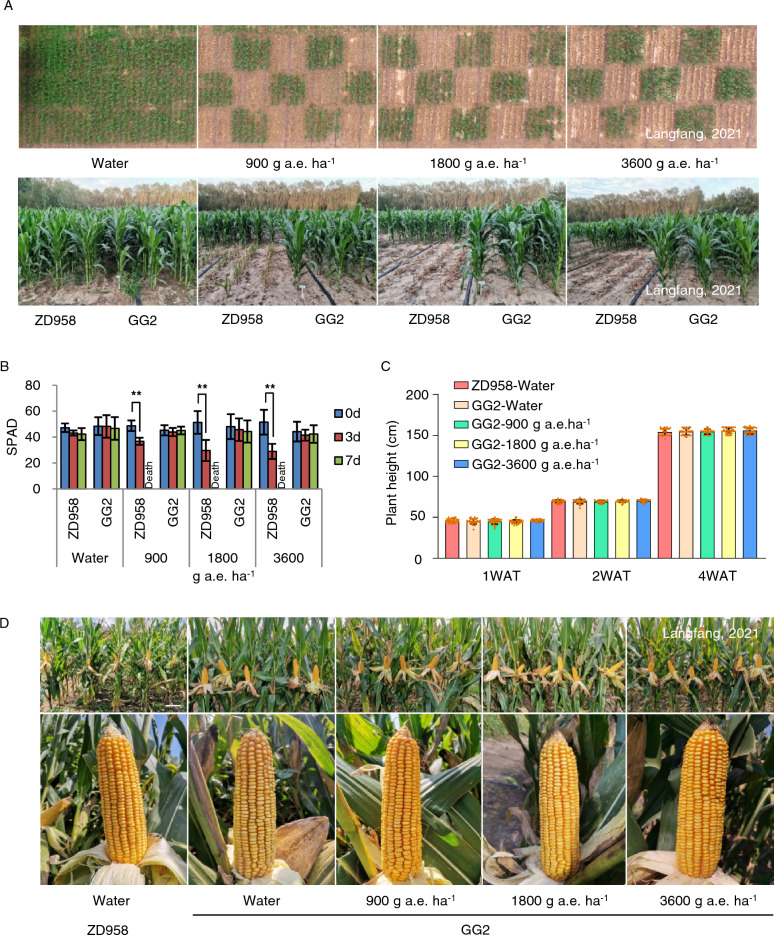
GR analysis of GG2 in the field (Langfang, Hebei Province, China) in 2021. **A** Photographs of GG2 and ZD958 (wild-type; WT) plants recorded at 4 weeks after treatment (WAT) with glyphosate (900, 1800, or 3600 g a.e. ha^–1^). Water was used as the negative control. Upper, GG2 maize and ZD958 were planted in a checkerboard pattern. Aerial view was taken by an unmanned aerial vehicle. Lower, GG2 maize growing normally after 900, 1800, and 3600 g a.e. ha^–1^ glyphosate treatment, with ZD958 only growing normally when treated with water. **B** Chlorophyll contents in GG2 and ZD958 leaves at 0, 3, and 7 days after glyphosate treatment (DAT). Data are means ± SD (*n* = 6). **P < 0.01, determined using a one-way ANOVA. **C** Plant height of GG2 at 1, 2, and 4 WAT with glyphosate. Data are means ± SD (*n* = 33). *P* > 0.05, determined using a one-way ANOVA. **D** Ear phenotype of ZD958 and GG2

The recommended field standard dose for glyphosate is 900 g a.e. ha^–1^ in a single application; however, there is a risk of maize being exposed to higher than intended doses due to spray overlaps or misapplications in the field. Commercial glyphosate-resistant crops must therefore tolerate glyphosate levels higher than the recommended dose. To evaluate the glyphosate resistance of the transgenic maize event GG2, we conducted a field experiment with applications of 900, 1800, or 3600 g a.e. ha^–1^ glyphosate at the four- to six-leaf stage. We used water only as the negative control, and ZD958 as the non-transgenic control. We recorded the seedling survival rate, plant height, and injury symptoms at 1, 2, and 4 weeks after treatment (WAT) with glyphosate. We also measured the chlorophyll content at 0, 3, and 7 days after glyphosate treatments (DAT) to monitor leaf development. The leaves of ZD958 gradually turned yellow and wilted after being sprayed with glyphosate, and their chlorophyll content significantly decreased in a dose-dependent pattern. After seven days, all ZD958 plants were dead at any dose of glyphosate, whereas we noticed no visible injury to GG2 plants following the same glyphosate treatments, with no significant change in their leaf chlorophyll content. The plant height and seedling survival rate of glyphosate-treated GG2 plants were not significantly different from those treated with water only at 1, 2, or 4 WAT (Fig. 5A–C and Table S3). Importantly, the agronomic traits of GG2 plants treated with glyphosate, such as plant height, ear height, ear length, ear diameter, row numbers per ear, kernels per row, hundred-kernel weight, and yield per plant, were not significantly different from those of ZD958 treated with water only at the harvesting stage (Fig. 5D and Table S4). This observation indicates that the transgenic maize event GG2 can tolerate four times the recommended glyphosate dose with no negative effect on yield, making it suitable for agricultural applications.

We conducted another field trial to explore the maximum glyphosate dose to which GG2 was resistant. Accordingly, we applied glyphosate doses of 1800, 3600, 5400, 7200, 9000, 10,800, 12,600, or 14,400 g a.e. ha^–1^ to GG2 plants at the four- to six-leaf stage. Although all GG2 plants survived even when treated with the highest dose of glyphosate, the height of GG2 plants decreased significantly when the glyphosate dose was 10,800 g a.e. ha^–1^ at 1, 2, and 4 WAT (Figure S5a–d). When the observation period was extended to harvest, plant height was no longer inhibited by high doses of glyphosate, and the GG2 plants were able to recover and exhibited a normal phenotype, especially in terms of kernels per row, ear length, hundred-kernel weight, and yield per plant (Figure S5e–l). These findings suggest that transgenic maize GG2 can tolerate ten times the recommended application dose of glyphosate in the field.

### Measurement of glyphosate residues in transgenic maize GG2

**Fig. 6:**
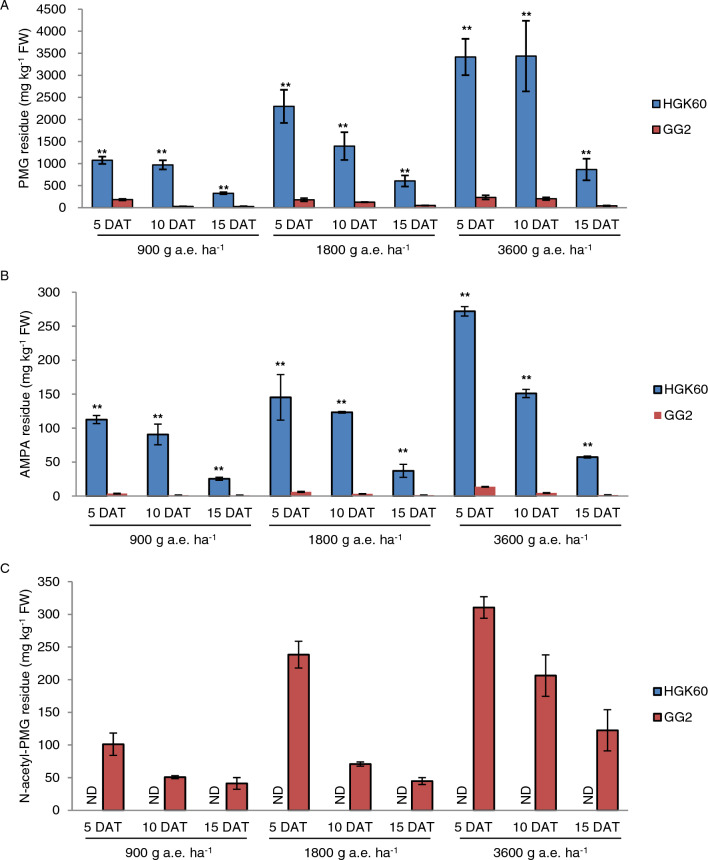
Glyphosate and glyphosate metabolites residue analysis in GG2 plants. In the field, GG2 plants at the four- to six-leaf stage were sprayed with 900, 1800, or 3600 g a.e. ha^–1^ glyphosate, using another glyphosate-resistant maize event (HGK60) as control. **A**–**C** PMG, AMPA, and *N*-acetyl-PMG residues in HGK60 and GG2 leaves at 5, 10, and 15 DAT with different doses of glyphosate. In **A**–**C**, data are means ± SD (*n* = 3 biological replications). *ND* not detected. **P < 0.01, determined using a one-way ANOVA

To assess whether the GAT enzyme can quickly metabolize glyphosate in transgenic maize GG2, we measured the residues of glyphosate (PMG), AMPA, and N-acetyl-PMG, using gas chromatography–mass spectrometry (GC–MS). We detected residues of PMG, AMPA, and *N*-acetyl-PMG in GG2 leaves after the application of 900, 1800, and 3600 g a.e. ha^–1^ glyphosate at 5 DAT to 15 DAT (Fig. 6A–C); however, the PMG and AMPA contents in GG2 leaves were 83%–97% and 95%–99% lower, respectively, than those of HGK60 leaves harboring the *g2-epsps* gene (Fig. 6A and B). This result indicates that the foreign GAT enzyme in GG2 plants contributes to the acetylation of PMG to N-acetyl-PMG, thereby reducing the amounts of PMG and AMPA residues. Taking into account the sum of PMG, AMPA, and N-acetyl-PMG contents as total glyphosate residues, these combined residues were 80%–92% lower in GG2 leaves than in HGK60 leaves. We also measured the glyphosate residues in silage maize and mature seeds and detected no glyphosate or glyphosate-metabolite residues in GG2 samples, even when the application of glyphosate was far higher than the field usage dose (Table S5). These glyphosate residues in transgenic maize GG2 conform to the national standard and pose no health risks.

## Discussion

Weeds significantly reduce the yield and quality of crops. Glyphosate is the most widely used herbicide in the world for weed control in crop fields; thus, the development of glyphosate-resistant crops and their use alongside glyphosate is an economical approach to weed management. The United States of America was the first country to cultivate commercial GM crops. According to reports from the U.S. Department of Agriculture (USDA), 90% of the maize grown in the USA is herbicide-resistant (USDA 2022), with most commercial glyphosate-resistant maize, such as NK603 (Monsanto; *cp4-epsps*), GA21 (Monsanto; *mepsps*), and 98,140 (DuPont; *gat4621*), using either *epsps* genes or *gat* genes. In recent years, the Chinese government has issued safety certificates for herbicide-resistant transgenic maize, which were mostly transformed with *cp4-epsps-like* or *mepsps* genes. *Gr79-epsps* and *gat* genes are new GR genes isolated from a glyphosate-polluted soil metagenomic library (Dun et al. 2006; Jin et al. 2007). Previous studies have confirmed that plants expressing *gr79-epsps* or *gat* had a high level of GR, with resistance similar to that of plants expressing *cp4-epsps* or *gat4621* (Heck et al. 2005; Liu et al. 2015; Ren et al. 2015). In this study, we stacked the *gr79-epsp*s and *gat* genes to obtain maize with high GR, indicating the excellent application value of *gr79-epsp*s and *gat* genes for the development of highly glyphosate-resistant crops.

The combination of two herbicide-tolerance genes with different mechanisms of action in the development of glyphosate-resistant crops has previously been shown to be an effective approach. Transgenic soybean expressing *g2-epsps* and *gat* genes could tolerate glyphosate up to 3600 g a.e. ha^–1^, and was certified safe for commercial planting (Guo et al. 2015). Transgenic cotton expressing *gr79-epsps* and *gat* genes resulted in higher resistance to glyphosate than plants expressing either gene alone (Liang et al. 2017). In this study, we generated transgenic maize plants co-expressing the *gr79-epsps* and *gat* genes via *Agrobacterium*-mediated transformation. Through field screening, molecular identification, and glyphosate tests, we obtained a single-copy insertion transgenic maize event, GG2, which possesses clear molecular characteristics, genetic stability, and high GR. We introgressed the transgenic maize event GG2 to the elite maize inbred lines Zheng58, Chang7-2, PH6WC, PH4CV, lx088, lx03-2, with no significant differences in agronomic traits compared to their parents. We are currently applying for a safety certificate for transgenic maize event GG2 to be used commercially in China (Liang et al. 2022). We believe it will have important uses both as an herbicide-resistant maize to control weeds, and as a refuge for insect- and herbicide-resistant maize to delay the development of pest resistance.

Generally, glyphosate is considered an environmentally friendly herbicide with no known risks to human or animal health (Duke and Powles 2008). It has a relatively short half-life in soil due to microbial degradation, ranging from a few days to months (Duke 2011); however, glyphosate metabolism in crops is slow, and it can be detected in the leaves and seeds of glyphosate-resistant soybean treated with labeled glyphosate (Duke 2011). To ensure safety for human and animal health, international and national institutions have set different maximum residue limit (MRL) standards for glyphosate and its metabolites, which in maize kernels is 5 mg kg^–1^ in the USA (and also in FAO/WHO recommendations), 1 mg kg^–1^ in the European Union, 3 mg kg^–1^ in Canada (Vicini et al. 2021; Xu et al. 2019), and 1 mg kg^–1^ in China (National Standard ID: GB2763-2021). GAT can quickly acetylate glyphosate to *N*-acetyl-glyphosate, which is not herbicidal (Green et al. 2008). GAT may therefore be a better choice for the development of a second wave of glyphosate-resistant crops (Duke 2011). In this study, we analyzed the residue of glyphosate and its metabolites in GG2 leaves at 5, 10, and 15 DAT with 900, 1800, or 3600 g a.e. ha^–1^ of glyphosate, revealing that the PMG and AMPA residues in GG2 leaves were 83%–97% lower than those of HGK60 leaves harboring *g2-epsps* gene. Both *g2-epsps* gene and *gr79-epsps* (also named *am79-epsps*) were cloned from the bacteria grown in soil heavily contaminated and belonged to Class I (Cao et al. 2012). The two genes’ function is the same. Although we detected *N*-acetyl-glyphosate residue in GG2, the total content of PMG, AMPA, and *N*-acetyl-glyphosate in GG2 plants was 80%–92% lower than in the control. The agronomic traits of GG2 maize were not affected by the application of glyphosate at any stage in its growth period, and we detected no traces of glyphosate or its metabolic residues (AMPA and *N*-acetyl-glyphosate) in silage maize or mature seeds. Transgenic maize event GG2, with its high GR and low residue content, therefore provides a valuable germplasm resource for maize breeding.

## Materials and methods

### Codon optimization and construction of the *gat *and* gr79-epsps* co-expression vector

To facilitate the efficient expression of the bacterial genes *gat* and *gr79-epsps* in maize, the codons of the *gat* and *gr79-epsps* genes were optimized according to OptimumGene, an algorithm developed by the GenScript Biotech Corporation (Nanjing, China). This step included the use of the maize preferential codons, increasing the GC content, and removing cryptic splicing sites. The resulting codon-optimized *gat* and *gr79-epsps* genes were synthesized by GenScript Biotech Corporation. To enhance the gene expression and guide the *gr79-epsps* gene into the maize chloroplasts, a 72-bp OMK sequence consisting of an Omega enhancer and Kozak motif was added upstream of the *gat* gene, and a 72-bp OMK sequence and a 141-bp sequence encoding a chloroplast peptide signal (CPS) from the maize ribulose bisphosphate carboxylase small subunit (RUBP) were added upstream of the *gr79-epsps* gene. The *OMK*-*gat* gene was inserted into an XhoI-digested binary vector, pCAMBIA2300, by seamless assembly cloning (Thermo Fisher Scientific, A14603), using the CaMV 35S promoter and a CaMV 35S polyA terminator (pCGAT). The *OMK:CPS-gr79* gene was digested by BamHI (5′) and KpnI (3′), and then inserted into a BamHI/KpnI-digested intermediate vector, pUC57-UN, which was modified from pUC57, using a *Ubiquitin* promoter from maize and *nos* terminator. Subsequently, the *gr79-epsps* expression cassette was digested by HindIII (5′) and EcoRI (3′) and ligated into pCGAT; the resulting plant expression vector was named pCGG.

### Maize transformation and elite inbred line backcrossing

The pCGG vector was transformed into immature zygotic maize B104 embryos using *Agrobacterium* (*Agrobacterium tumefaciens*) strain EHA105. *Agrobacterium*-mediated transformation and regeneration of transgenic plants were conducted as previously described (Frame et al. 2002). Positive calli and regenerated plantlets were selected on Murashige and Skoog (MS) regeneration medium containing glyphosate (0.2 g L^–1^). The T_0_ generation maize plants were self-pollinated and the T_1_ seeds were harvested. Continuous outcrossing to the elite inbred lines Zheng58 and Chang7-2 was performed for six generations from the T_1_ generation, and the resulting offspring were self-pollinated for three generations to introduce the homozygous transgenic event in the Zheng58 and Chang7-2 backgrounds. The presence of the transgenic event was confirmed with molecular detection and glyphosate screening.

### PCR and Southern blot analysis

Genomic DNA was isolated from fresh leaf tissues using the CTAB method (Porebski et al. 1997). The primers of the *gat* and *gr79-epsps* genes are listed in Table S1. The 20-μL PCR mixtures contained 100 ng of DNA template, 0.1 μM of each primer, and 10 μL 2 × Taq MasterMix (Dye plus) (Vazyme, Nanjing, China). The PCR products were visualized with gel-red and documented on G:BOX BioImaging systems (SYNGENE, Cambridge, UK). About 100 μg of genomic DNA was used for the Southern blot analysis, according to previously reported methods (Li et al. 2018). The genomic DNA was digested with HindIII or KpnI (New England Biolabs, Ipswich, Massachusetts, USA). A 249-bp fragment of the *gat* gene, an 831-bp fragment of the *gr79-epsps* gene, a 425-bp fragment of the 35S promoter, a 181-bp fragment of the CaMV polyA terminator, a 1036-bp fragment of the *Ubiquitin* promoter, a 371-bp fragment of the *nos* terminator, and 2291-bp, 2230-bp, and 2233-bp fragments of the vector backbone, were used as probes in the Southern blot analysis. Each probe was amplified from pCGG by PCR using the primers listed in Table S2.

### End-point RT-PCR and RT-qPCR analysis

Total RNA was isolated from different maize tissues (leaf, root, stem, pollen, tassel, silk, ear, and seed) at different stages (V1, V6, R1, and R6) (Abendroth et al. 2011) using Trizol reagent (Vazyme, Nanjing, China). Approximately 1 μg of total RNA was used as a template for reverse transcription with a HiScript III 1st Strand cDNA Synthesis Kit (+ gDNA wiper) (Vazyme), according to the manufacturer’s instructions. Maize *Actin1* was used as a reference gene. The 20-μL reaction mixtures for end-point RT-PCR contained 10 μL 2 × Taq MasterMix (Dye plus) (Vazyme), 0.1 μM of each primer, and 1 μL of cDNA template. The 20-μL reaction mixtures of RT-qPCR contained 10 μL 2 × Taq Pro Universal SYBR qPCR Master Mix (Vazyme), 0.2 μM of each primer, and 1 μL of the cDNA template. qPCR was performed with 2^ΔΔCt^ method quantification using an ABI QuantStudio 3 instrument (Thermo Fisher Scientific, Waltham, Massachusetts, USA). Primers used in the end-point RT-PCR and RT-qPCR are listed in Table S2.

### ELISA

ELISA kits for the quantitative detection of GAT and GR79-EPSPS (YouLong Biotech, Shanghai, China) were used to examine total proteins extracted from different maize tissues (leaf, root, stem, pollen, tassel, silk, ear, and seed) at different stages (V1, V6, R1, and R6), following the manufacturer’s protocol. The optical density (OD) of the samples was measured at 450 nm and 630 nm using a BioTek Elx808 plate reader (BioTek Instruments, Winooski, Vermont, USA).

### T-DNA insertion site, event-specific PCR, and full-length foreign insertion sequence analysis

The T-DNA-flanking sequences in transgenic maize plants were extended using the TAIL-PCR method with a Genome Walking Kit (TaKaRa Bio, Tokyo, Japan). The left border (LB)-flanking maize DNA sequence was used with three nested specific primers (GAT-SP1, GAT-SP2, and GAT-SP4) and combined with a degenerate primer, AP4, provided in the Genome Walking Kit. All experimental procedures were carried out according to the manufacturer’s instructions. The 3rd PCR product was isolated from 0.8% Agarose gel and Sanger sequenced. The product sequence was analyzed using a BLAST sequence alignment against MaizeGDB (https://www.maizegdb.org, B73 RefGen_V4) to identify the T-DNA insertion site. Two pairs of specific primers (P1/P2, P3/P4) were synthesized to amplify the extension of the LB-flanking and right border (RB)-flanking sequences of GG2, including both the maize genome sequence and the foreign insertion sequence. This specific PCR detection method was used to test the transgenic maize event GG2. According to the reference T-DNA sequence and GG2-flanking maize genome sequence, four pairs of PCR primers (frag1-F/R, frag2-F/R, frag3-F/R, frag4-F/R) were synthesized to amplify the full-length foreign insertion sequence, which was ligated into the pEASY-Blunt Cloning Vector (TransGen Biotech, Beijing, China) for Sanger sequencing. The primers used for TAIL-PCR, event-specific PCR, and PCR are listed in Table S2.

### Target herbicide-tolerance analysis

Commercially formulated isopropylamine salt of glyphosate was used at a concentration of 300 g a.e. L^−1^ (Roundup; Bayer, Leverkusen, Germany) to assess the target herbicide (glyphosate) resistance levels of the transgenic maize plants. The medium recommended dose for glyphosate application is 900 g a.e. ha^–1^, according to the manufacturer’s manual. Glyphosate was applied to maize plants in the field at dosages of 900, 1800, and 3600 g a.e. ha^–1^ to select highly glyphosate-resistant plants and assess the resistance levels to the herbicide. Doses of 5400, 7200, 9000, 10,800, 12,600, and 14,400 g a.e. ha^–1^ were used to determine the maximum GR dose in the field. The survival rate, plant height, and injury symptoms of all plants were recorded at 1, 2, and 4 weeks after treatment (WAT). The agronomic traits were investigated and recorded at the R6 stage.

### Chlorophyll content analysis

ZD958 and GG2 plants were sprayed with different doses of glyphosate (900, 1800, or 3600 g a.e. ha^–1^) at the four- to six-leaf stage, using water as the negative control. The chlorophyll contents of the sixth leaves were measured with a SPAD-502 Plus chlorophyll meter (Konica Minolta, Tokyo, Japan) at 0, 3, and 7 days after treatment (DAT).

### Agronomic traits investigation

The agronomic traits of maize (plant height, ear height, ear length, ear diameter, row numbers per ear, kernels per row, bald tip length, hundred-kernel weight, and yield per plant), were investigated and analyzed.

### Quantification of glyphosate and glyphosate-metabolite residues

In the field, GG2 plants were sprayed at the four- to six-leaf stage with different doses of glyphosate, using the transgenic maize event HGK60 as a control. HGK60 is a transgenic maize glyphosate-resistant event developed by our group, which expresses a single *g2-epsps* gene (Liang et al. 2018). The sixth leaves were harvested at 5, 10, and 15 DAT. Silage maize plants and mature seeds were harvested at the R3 stage (80 DAT) and R6 stage (110 DAT), respectively. About 5 g leaf samples, 25 g silage plant samples, or 25 g seed samples were ground in liquid nitrogen. Three biological replications were performed for each treatment. The glyphosate residues in the powdered samples were measured according to the described previously (Yang et al. 2020). GC–MS was performed on a GC-QQQ 7000D (Agilent Technologies, Santa Clara, California, USA). An HP-5MS UI column (30.0 m long, 0.25-mm internal diameter, and 0.25-μm film thickness) was used as a chromatographic column. The contents of PMG, AMPA, and N-acetyl-PMG were determined using their standard curves, as shown in Figure S6a–c.

### Statistical analysis

The data of chlorophyll content were analyzed using SAS 9.4 (SAS, Cary, North Carolina, USA) and Excel (Microsoft Corporation, Redmond, Washington, USA). The data of agronomic traits were analyzed using GraphPad Prism 9.4 (GraphPad Software, Boston, Massachusetts, USA).

### Accession numbers

*ZmHAD*: Zm00001d033633, and *ZmActin1*: Zm00001d010159.

### Supplementary Information

Below is the link to the electronic supplementary material.

Supplementary file1 (PPTX 1740 KB)

Supplementary file2 (XLSX 17 KB)

## Data Availability

All data are available from the corresponding author upon reasonable request.
