# Supplementary material for: In maize, co-expression of GAT and GR79-EPSPS provides high glyphosate resistance, along with low glyphosate residues
Source: aBIOTECH. 2023 Sep 1;4(4):277–90. doi: 10.1007/s42994-023-00114-8 (PMC10721750; doi:10.1007/s42994-023-00114-8)
Supplement: Supplementary file 1 — Supplementary file1 (PPTX 1740 KB) [file 42994_2023_114_MOESM1_ESM.pptx]

## Slide 1
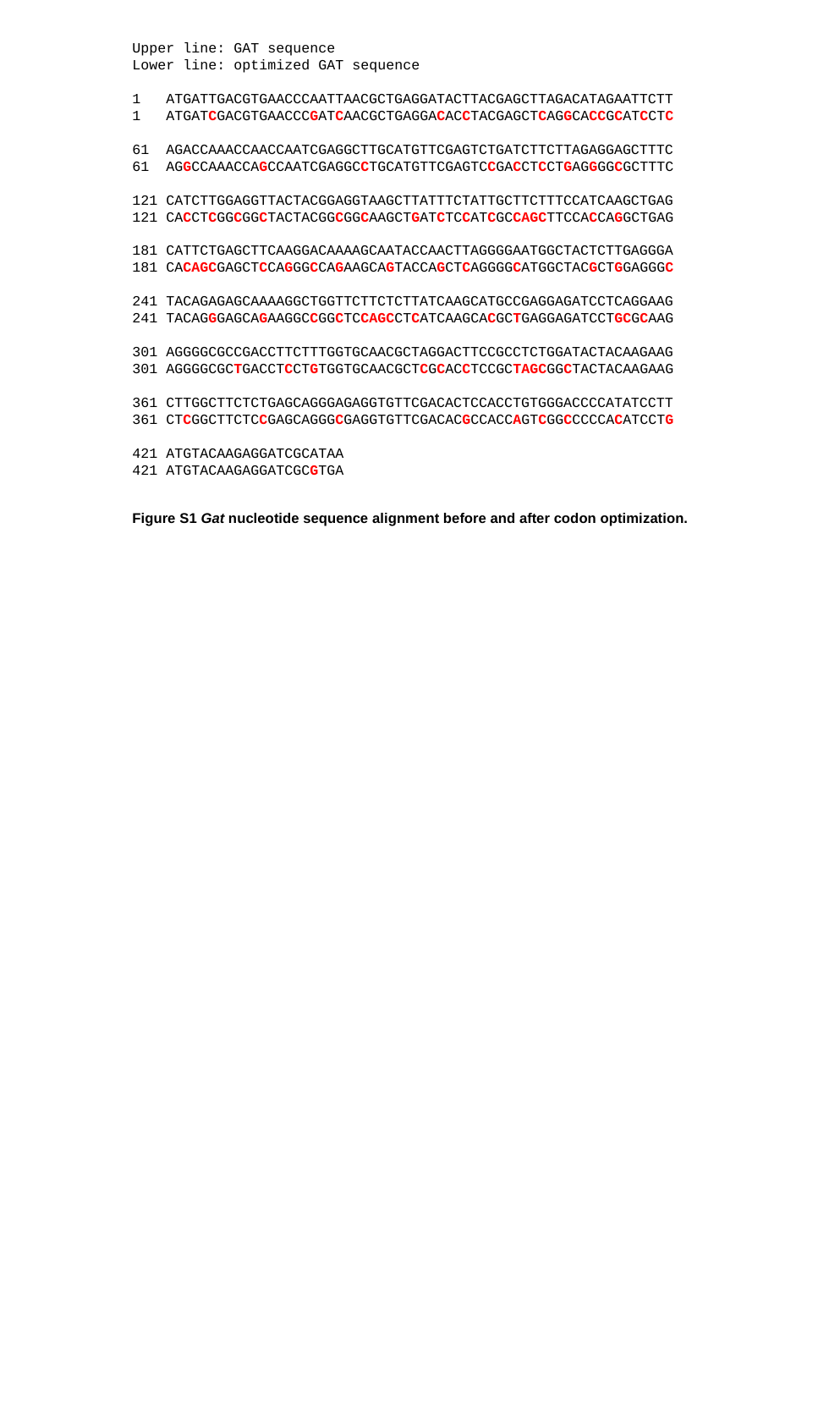

Upper line: GAT sequence
Lower line: optimized GAT sequence
1 ATGATTGACGTGAACCCAATTAACGCTGAGGATACTTACGAGCTTAGACATAGAATTCTT
1 ATGATCGACGTGAACCCGATCAACGCTGAGGACACCTACGAGCTCAGGCACCGCATCCTC
61 AGACCAAACCAACCAATCGAGGCTTGCATGTTCGAGTCTGATCTTCTTAGAGGAGCTTTC
61 AGGCCAAACCAGCCAATCGAGGCCTGCATGTTCGAGTCCGACCTCCTGAGGGGCGCTTTC
121 CATCTTGGAGGTTACTACGGAGGTAAGCTTATTTCTATTGCTTCTTTCCATCAAGCTGAG
121 CACCTCGGCGGCTACTACGGCGGCAAGCTGATCTCCATCGCCAGCTTCCACCAGGCTGAG
181 CATTCTGAGCTTCAAGGACAAAAGCAATACCAACTTAGGGGAATGGCTACTCTTGAGGGA
181 CACAGCGAGCTCCAGGGCCAGAAGCAGTACCAGCTCAGGGGCATGGCTACGCTGGAGGGC
241 TACAGAGAGCAAAAGGCTGGTTCTTCTCTTATCAAGCATGCCGAGGAGATCCTCAGGAAG
241 TACAGGGAGCAGAAGGCCGGCTCCAGCCTCATCAAGCACGCTGAGGAGATCCTGCGCAAG
301 AGGGGCGCCGACCTTCTTTGGTGCAACGCTAGGACTTCCGCCTCTGGATACTACAAGAAG
301 AGGGGCGCTGACCTCCTGTGGTGCAACGCTCGCACCTCCGCTAGCGGCTACTACAAGAAG
361 CTTGGCTTCTCTGAGCAGGGAGAGGTGTTCGACACTCCACCTGTGGGACCCCATATCCTT
361 CTCGGCTTCTCCGAGCAGGGCGAGGTGTTCGACACGCCACCAGTCGGCCCCCACATCCTG
421 ATGTACAAGAGGATCGCATAA
421 ATGTACAAGAGGATCGCGTGA
Figure S1 Gat nucleotide sequence alignment before and after codon optimization.

## Slide 2
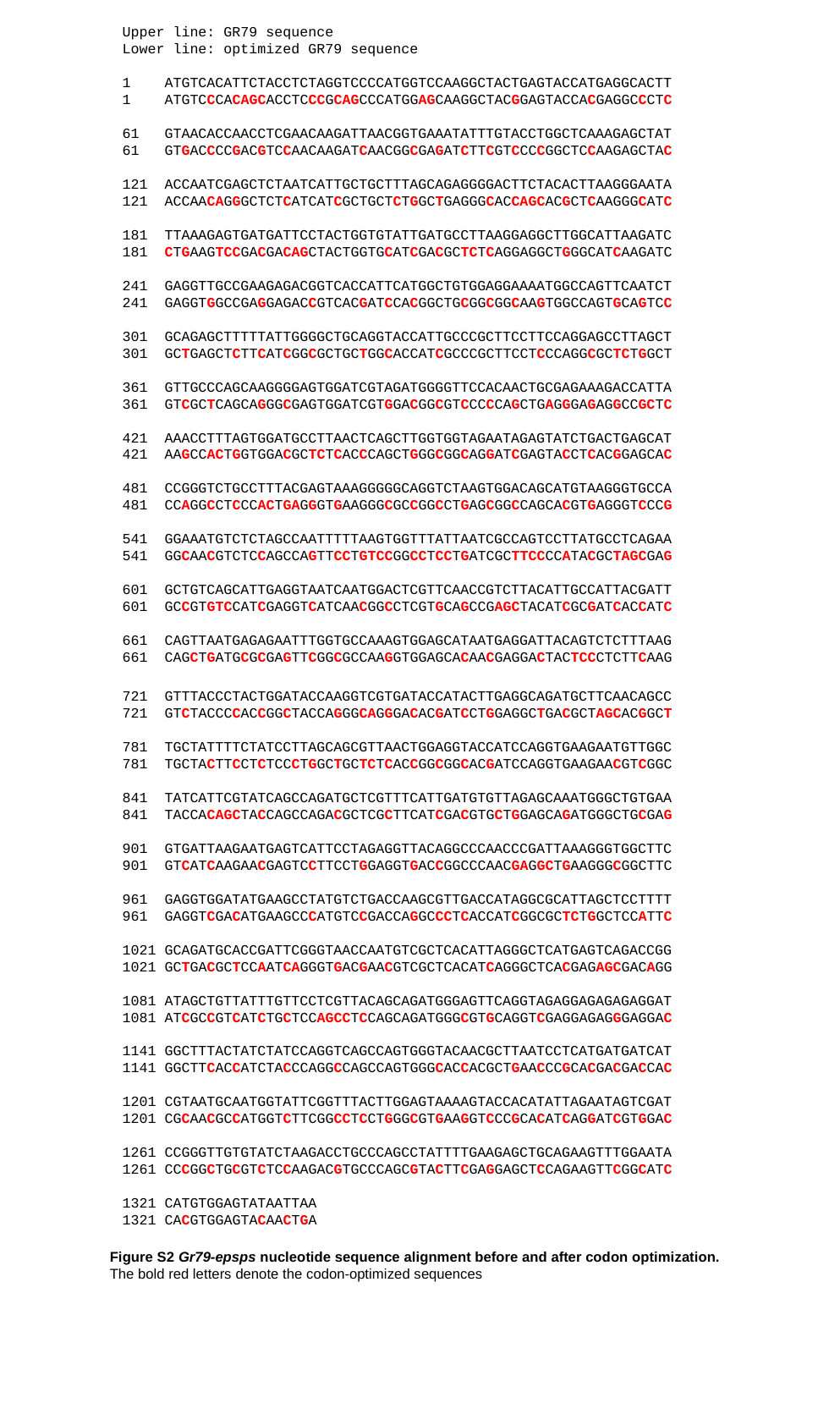

Upper line: GR79 sequence
Lower line: optimized GR79 sequence
1 ATGTCACATTCTACCTCTAGGTCCCCATGGTCCAAGGCTACTGAGTACCATGAGGCACTT
1 ATGTCCCACAGCACCTCCCGCAGCCCATGGAGCAAGGCTACGGAGTACCACGAGGCCCTC
61 GTAACACCAACCTCGAACAAGATTAACGGTGAAATATTTGTACCTGGCTCAAAGAGCTAT
61 GTGACCCCGACGTCCAACAAGATCAACGGCGAGATCTTCGTCCCCGGCTCCAAGAGCTAC
121 ACCAATCGAGCTCTAATCATTGCTGCTTTAGCAGAGGGGACTTCTACACTTAAGGGAATA
121 ACCAACAGGGCTCTCATCATCGCTGCTCTGGCTGAGGGCACCAGCACGCTCAAGGGCATC
181 TTAAAGAGTGATGATTCCTACTGGTGTATTGATGCCTTAAGGAGGCTTGGCATTAAGATC
181 CTGAAGTCCGACGACAGCTACTGGTGCATCGACGCTCTCAGGAGGCTGGGCATCAAGATC
241 GAGGTTGCCGAAGAGACGGTCACCATTCATGGCTGTGGAGGAAAATGGCCAGTTCAATCT
241 GAGGTGGCCGAGGAGACCGTCACGATCCACGGCTGCGGCGGCAAGTGGCCAGTGCAGTCC
301 GCAGAGCTTTTTATTGGGGCTGCAGGTACCATTGCCCGCTTCCTTCCAGGAGCCTTAGCT
301 GCTGAGCTCTTCATCGGCGCTGCTGGCACCATCGCCCGCTTCCTCCCAGGCGCTCTGGCT
361 GTTGCCCAGCAAGGGGAGTGGATCGTAGATGGGGTTCCACAACTGCGAGAAAGACCATTA
361 GTCGCTCAGCAGGGCGAGTGGATCGTGGACGGCGTCCCCCAGCTGAGGGAGAGGCCGCTC
421 AAACCTTTAGTGGATGCCTTAACTCAGCTTGGTGGTAGAATAGAGTATCTGACTGAGCAT
421 AAGCCACTGGTGGACGCTCTCACCCAGCTGGGCGGCAGGATCGAGTACCTCACGGAGCAC
481 CCGGGTCTGCCTTTACGAGTAAAGGGGGCAGGTCTAAGTGGACAGCATGTAAGGGTGCCA
481 CCAGGCCTCCCACTGAGGGTGAAGGGCGCCGGCCTGAGCGGCCAGCACGTGAGGGTCCCG
541 GGAAATGTCTCTAGCCAATTTTTAAGTGGTTTATTAATCGCCAGTCCTTATGCCTCAGAA
541 GGCAACGTCTCCAGCCAGTTCCTGTCCGGCCTCCTGATCGCTTCCCCATACGCTAGCGAG
601 GCTGTCAGCATTGAGGTAATCAATGGACTCGTTCAACCGTCTTACATTGCCATTACGATT
601 GCCGTGTCCATCGAGGTCATCAACGGCCTCGTGCAGCCGAGCTACATCGCGATCACCATC
661 CAGTTAATGAGAGAATTTGGTGCCAAAGTGGAGCATAATGAGGATTACAGTCTCTTTAAG
661 CAGCTGATGCGCGAGTTCGGCGCCAAGGTGGAGCACAACGAGGACTACTCCCTCTTCAAG
721 GTTTACCCTACTGGATACCAAGGTCGTGATACCATACTTGAGGCAGATGCTTCAACAGCC
721 GTCTACCCCACCGGCTACCAGGGCAGGGACACGATCCTGGAGGCTGACGCTAGCACGGCT
781 TGCTATTTTCTATCCTTAGCAGCGTTAACTGGAGGTACCATCCAGGTGAAGAATGTTGGC
781 TGCTACTTCCTCTCCCTGGCTGCTCTCACCGGCGGCACGATCCAGGTGAAGAACGTCGGC
841 TATCATTCGTATCAGCCAGATGCTCGTTTCATTGATGTGTTAGAGCAAATGGGCTGTGAA
841 TACCACAGCTACCAGCCAGACGCTCGCTTCATCGACGTGCTGGAGCAGATGGGCTGCGAG
901 GTGATTAAGAATGAGTCATTCCTAGAGGTTACAGGCCCAACCCGATTAAAGGGTGGCTTC
901 GTCATCAAGAACGAGTCCTTCCTGGAGGTGACCGGCCCAACGAGGCTGAAGGGCGGCTTC
961 GAGGTGGATATGAAGCCTATGTCTGACCAAGCGTTGACCATAGGCGCATTAGCTCCTTTT
961 GAGGTCGACATGAAGCCCATGTCCGACCAGGCCCTCACCATCGGCGCTCTGGCTCCATTC
1021 GCAGATGCACCGATTCGGGTAACCAATGTCGCTCACATTAGGGCTCATGAGTCAGACCGG
1021 GCTGACGCTCCAATCAGGGTGACGAACGTCGCTCACATCAGGGCTCACGAGAGCGACAGG
1081 ATAGCTGTTATTTGTTCCTCGTTACAGCAGATGGGAGTTCAGGTAGAGGAGAGAGAGGAT
1081 ATCGCCGTCATCTGCTCCAGCCTCCAGCAGATGGGCGTGCAGGTCGAGGAGAGGGAGGAC
1141 GGCTTTACTATCTATCCAGGTCAGCCAGTGGGTACAACGCTTAATCCTCATGATGATCAT
1141 GGCTTCACCATCTACCCAGGCCAGCCAGTGGGCACCACGCTGAACCCGCACGACGACCAC
1201 CGTAATGCAATGGTATTCGGTTTACTTGGAGTAAAAGTACCACATATTAGAATAGTCGAT
1201 CGCAACGCCATGGTCTTCGGCCTCCTGGGCGTGAAGGTCCCGCACATCAGGATCGTGGAC
1261 CCGGGTTGTGTATCTAAGACCTGCCCAGCCTATTTTGAAGAGCTGCAGAAGTTTGGAATA
1261 CCCGGCTGCGTCTCCAAGACGTGCCCAGCGTACTTCGAGGAGCTCCAGAAGTTCGGCATC
1321 CATGTGGAGTATAATTAA
1321 CACGTGGAGTACAACTGA
Figure S2 Gr79-epsps nucleotide sequence alignment before and after codon optimization. The bold red letters denote the codon-optimized sequences

## Slide 3
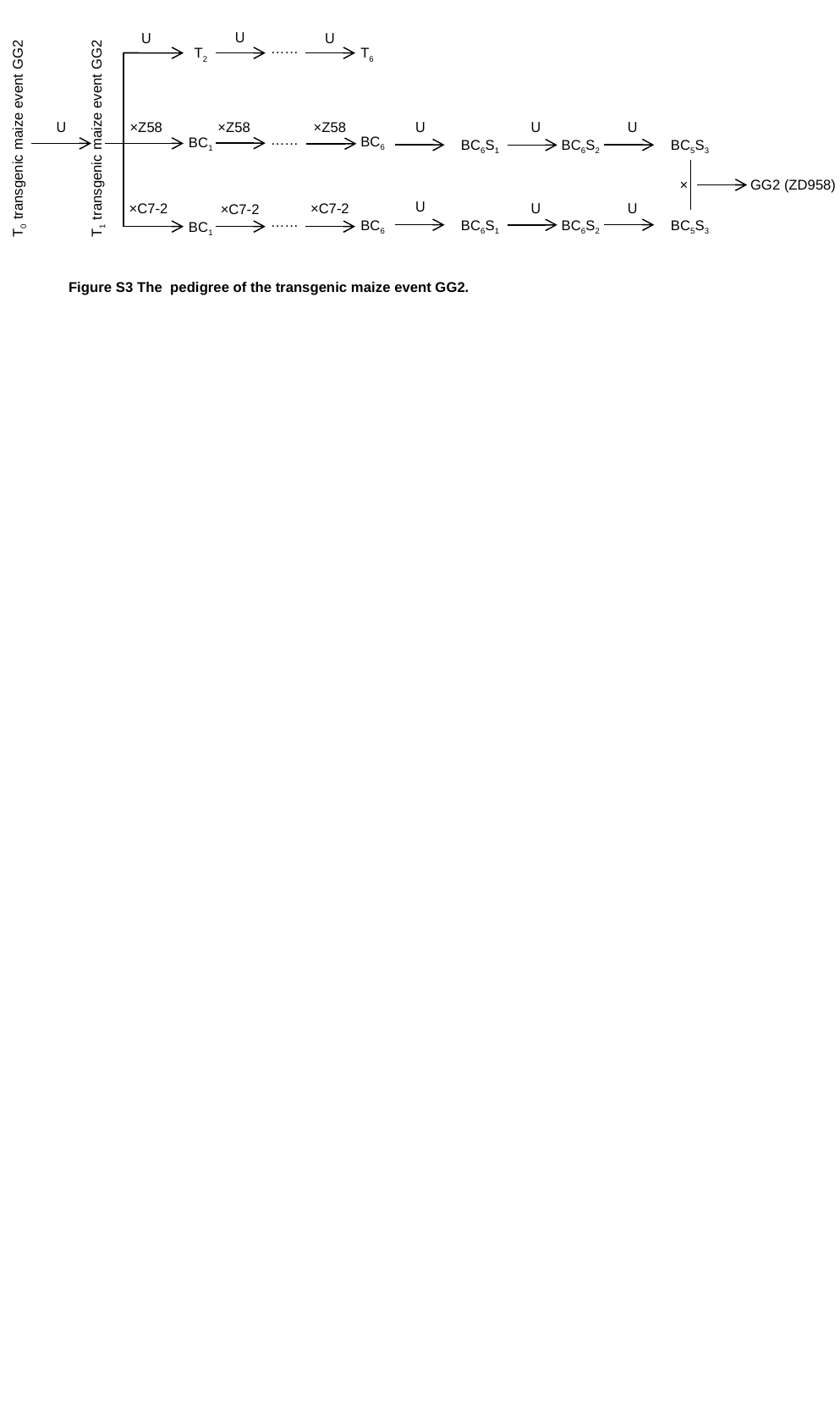

U
U
U
……
T6
T2
U
×Z58
×Z58
U
×Z58
U
U
T0 transgenic maize event GG2
T1 transgenic maize event GG2
……
BC6
BC1
BC6S1
BC5S3
BC6S2
×
GG2 (ZD958)
U
×C7-2
×C7-2
U
U
×C7-2
……
BC6
BC6S1
BC5S3
BC6S2
BC1
Figure S3 The pedigree of the transgenic maize event GG2.

## Slide 4
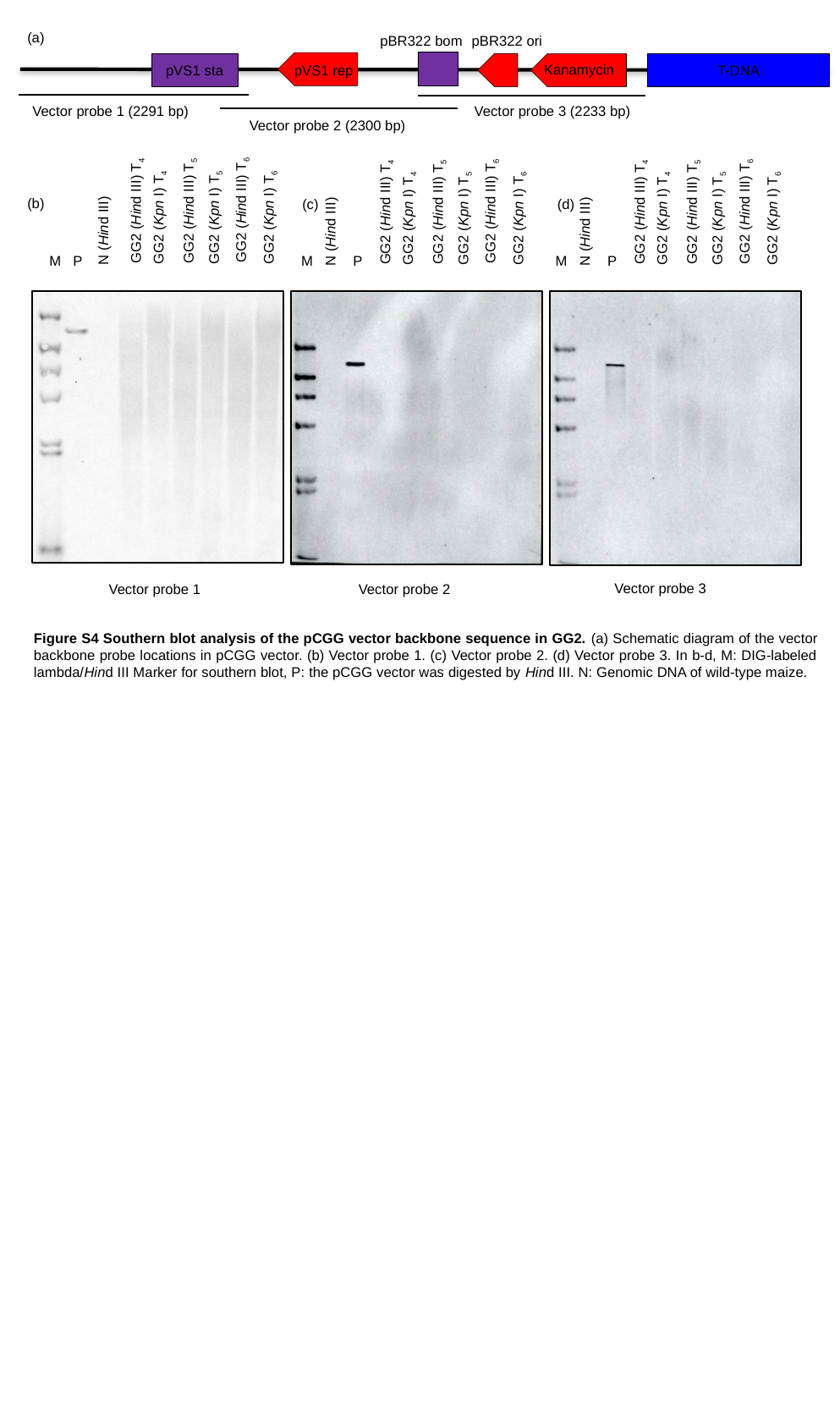

(a)
pBR322 bom
pBR322 ori
Kanamycin
T-DNA
pVS1 sta
pVS1 rep
Vector probe 1 (2291 bp)
Vector probe 3 (2233 bp)
Vector probe 2 (2300 bp)
GG2 (Hind III) T6
GG2 (Hind III) T4
GG2 (Hind III) T5
GG2 (Kpn I) T5
GG2 (Kpn I) T6
GG2 (Kpn I) T4
N (Hind III)
M P
GG2 (Hind III) T6
GG2 (Hind III) T4
GG2 (Hind III) T5
GG2 (Kpn I) T5
GG2 (Kpn I) T6
GG2 (Kpn I) T4
N (Hind III)
M P
Vector probe 2
GG2 (Hind III) T6
GG2 (Hind III) T4
GG2 (Hind III) T5
GG2 (Kpn I) T5
GG2 (Kpn I) T6
GG2 (Kpn I) T4
N (Hind III)
M P
Vector probe 3
(b)
(c)
(d)
Vector probe 1
Figure S4 Southern blot analysis of the pCGG vector backbone sequence in GG2. (a) Schematic diagram of the vector backbone probe locations in pCGG vector. (b) Vector probe 1. (c) Vector probe 2. (d) Vector probe 3. In b-d, M: DIG-labeled lambda/Hind III Marker for southern blot, P: the pCGG vector was digested by Hind III. N: Genomic DNA of wild-type maize.

## Slide 5
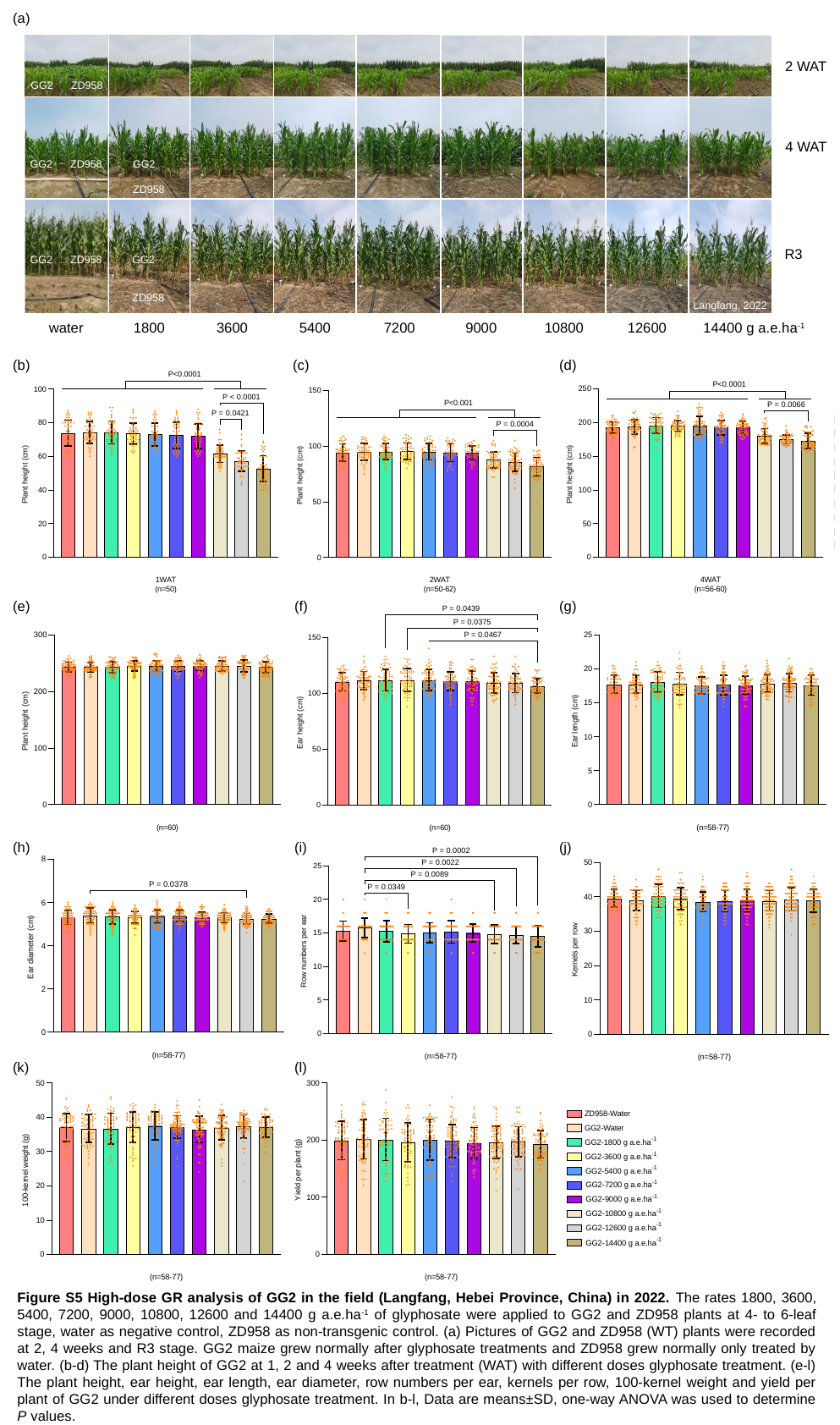

(a)
2 WAT
4 WAT
R3
10800
12600
14400 g a.e.ha-1
water
1800
3600
5400
9000
7200
GG2 ZD958
GG2 ZD958
GG2
ZD958
GG2 ZD958
GG2
ZD958
Langfang, 2022
(b)
(c)
(d)
(g)
(e)
(f)
(j)
(h)
(i)
(k)
(l)
Figure S5 High-dose GR analysis of GG2 in the field (Langfang, Hebei Province, China) in 2022. The rates 1800, 3600, 5400, 7200, 9000, 10800, 12600 and 14400 g a.e.ha-1 of glyphosate were applied to GG2 and ZD958 plants at 4- to 6-leaf stage, water as negative control, ZD958 as non-transgenic control. (a) Pictures of GG2 and ZD958 (WT) plants were recorded at 2, 4 weeks and R3 stage. GG2 maize grew normally after glyphosate treatments and ZD958 grew normally only treated by water. (b-d) The plant height of GG2 at 1, 2 and 4 weeks after treatment (WAT) with different doses glyphosate treatment. (e-l) The plant height, ear height, ear length, ear diameter, row numbers per ear, kernels per row, 100-kernel weight and yield per plant of GG2 under different doses glyphosate treatment. In b-l, Data are means±SD, one-way ANOVA was used to determine P values.

## Slide 6
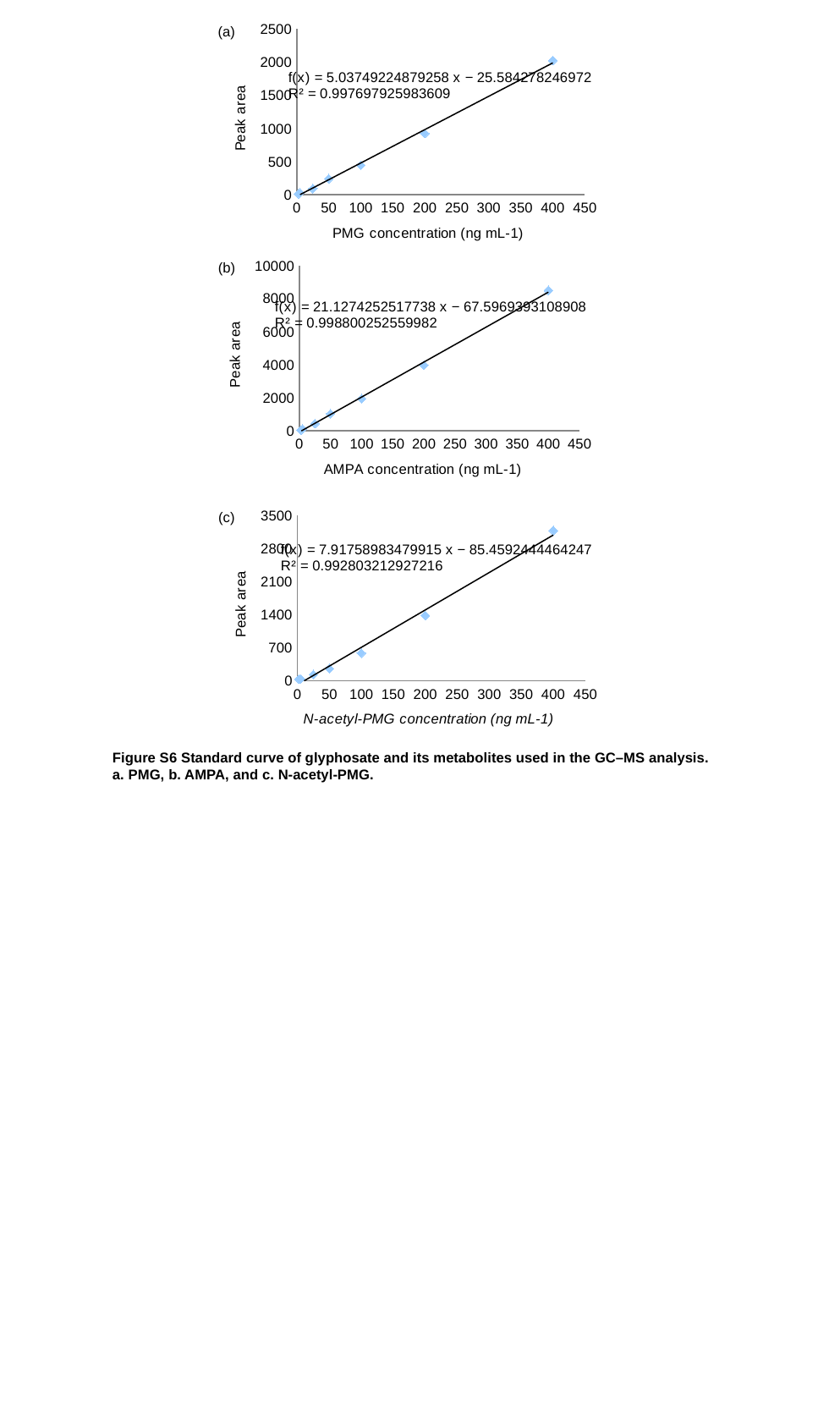

### Chart
| Category | PMG area |
|---|---|(a)
(b)
### Chart
| Category | AMPA area |
|---|---|(c)
### Chart
| Category | N-acetyl-PMG |
|---|---|Figure S6 Standard curve of glyphosate and its metabolites used in the GC–MS analysis. a. PMG, b. AMPA, and c. N-acetyl-PMG.
